# Cyclodextrins as Modulators of Gut Microbiota: Pharmaceutical Applications and Impact on Intestinal Health

**DOI:** 10.3390/pharmaceutics17060752

**Published:** 2025-06-07

**Authors:** Renata Maria Varut, Mircea Sorin Ciolofan, Maria Elena Veronica, Kristina Radivojević, Diana Maria Trasca, Cristina Popescu, Oana Diaconu, Cristina Elena Singer

**Affiliations:** 1 Research Methodology Department, Faculty of Pharmacy, University of Medicine and Pharmacy of Craiova, 200349 Craiova, Romania; renata.varut@umfcv.ro (R.M.V.); kristinaradivojevic03@gmail.com (K.R.); 2 Department of ENT, University of Medicine and Pharmacy of Craiova, 200349 Craiova, Romania; sorin.ciolofan@umfcv.ro; 3 Department of Mother and Baby, University of Medicine and Pharmacy of Craiova, 200349 Craiova, Romania; veronica.maria@umfcv.ro (M.E.V.); cristina.singer@umfcv.ro (C.E.S.); 4 Department of Internal Medicine, University of Medicine and Pharmacy of Craiova, 200349 Craiova, Romania; 5 Department of Anatomy, University of Medicine and Pharmacy, 200349 Craiova, Romania; 6 Department of Endodontics, University of Medicine and Pharmacy, 200349 Craiova, Romania; oana.diaconu@umfcv.ro

**Keywords:** cyclodextrins, gut microbiota, SCFAs, drug delivery systems, prebiotics, dysbiosis, pharmaceutical excipients

## Abstract

**Background/Objectives**: Cyclodextrins (CDs) have garnered increasing attention in pharmaceutical research due to their ability to enhance drug solubility, bioavailability, and therapeutic efficacy. Meanwhile, the gut microbiota, a key regulator of human health, has emerged as an important target in evaluating the safety and broader implications of pharmaceutical excipients. This review aims to synthesize current knowledge regarding the effects of CDs on the composition and function of the gut microbiota. **Methods**: A literature search following PRISMA guidelines was conducted in PubMed, ScienceDirect, and Google Scholar to identify studies on cyclodextrins and their interactions with gut microbiota. **Results**: Cyclodextrins, particularly α-, β-, and γ-CDs, demonstrated the capacity to modulate gut microbiota composition, promoting the growth of beneficial bacteria such as *Bifidobacterium* and *Akkermansia*. Supplementation with CDs was also associated with an increased production of short-chain fatty acids (SCFAs), which are essential for maintaining intestinal homeostasis and metabolic health. Moreover, CDs exhibited potential in lowering lipid levels and improving postprandial glycemic control without enhancing insulin secretion. Although generally recognized as safe, the toxicological profile of CDs varies depending on their type, dosage, and route of administration. **Conclusions**: Cyclodextrins hold considerable promise not only as pharmaceutical excipients but also as modulators of gut microbial communities, suggesting a dual therapeutic and prebiotic role. Future studies integrating metagenomic and metabolomic approaches are necessary to further elucidate the molecular mechanisms underlying CD–microbiota interactions and to optimize their application in enhancing drug delivery efficiency and promoting intestinal health.

## 1. Introduction

**Figure 1:**
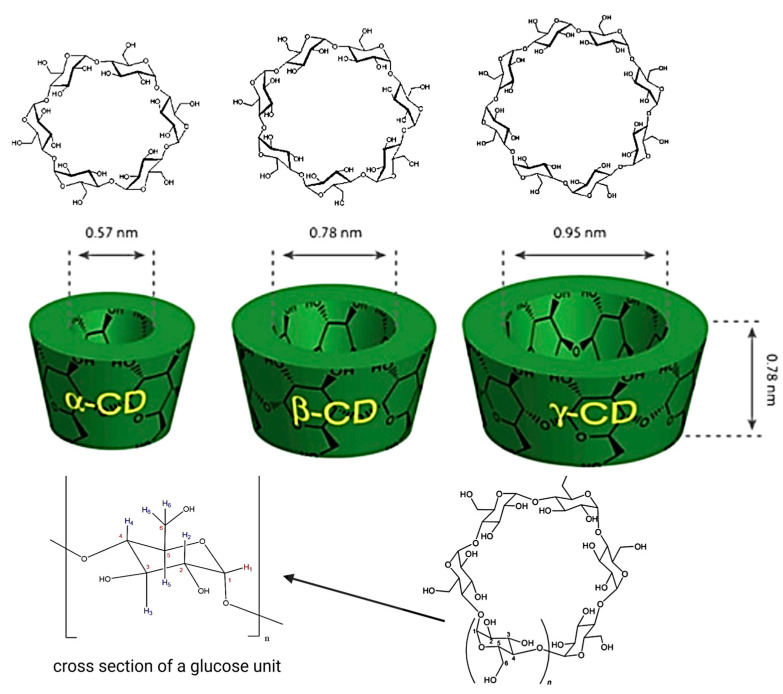
Structure of CDs. https://app.biorender.com/illustrations/6810dc6cb1ff952cb9d926eb (accessed on 14 May 2025).

Cyclodextrins (CDs) constitute a class of cyclic macromolecules formed from α-1,4-linked D-glucopyranose units, which are enzymatically derived from the hydrolysis of starch [1]. Their distinctive molecular architecture, often described as a truncated cone, presents a hydrophobic internal cavity and a hydrophilic external surface—an arrangement that underlies their ability to host various lipophilic molecules. Among the naturally occurring CDs, α-CD, β-CD, and γ-CD, comprising six, seven, and eight glucose residues, respectively, are the primary forms employed in pharmaceutical formulations due to their favorable physicochemical and safety profiles (Figure 1).

These cyclic oligosaccharides have become widely established as excipients in drug delivery, primarily because of their unique structural features, including chemically accessible hydroxyl groups, high water solubility, and the ability to form stable inclusion complexes with a wide variety of guest compounds [2,3,4]. Their incorporation into commercial medicinal products is extensive, and their safety and utility are recognized through inclusion in official pharmacopoeias and approval by regulatory bodies across multiple jurisdictions.

In recent years, research interest has expanded from conventional CD inclusion complexes to advanced CD-based nanocarriers. These systems offer enhanced pharmacological performance by increasing the bioavailability of poorly soluble drugs, facilitating targeted delivery to specific tissues, prolonging drug retention in systemic circulation, and supporting synergistic interactions between therapeutic agents [5,6]. Furthermore, CDs exhibit multifunctionality: they can reduce adverse effects such as gastrointestinal or ocular irritation, improve the palatability of oral formulations, prevent chemical incompatibilities, and even stabilize volatile or sensitive compounds [7]. Their versatility also extends to formulation engineering, where they are used to convert oily or liquid ingredients into powder form for improved handling and dosing accuracy.

For certain therapeutics, particularly those with unpleasant tastes or odors, palatability poses a significant barrier to patient adherence, especially in children. Cyclodextrins help address this challenge by encapsulating the offending molecules, effectively neutralizing their undesirable organoleptic properties and improving treatment compliance [8]. Simultaneously, the last decade has witnessed an explosion in microbiome research, largely driven by advances in high-throughput sequencing technologies. These efforts have yielded comprehensive databases of the human gut microbiota, deepening our understanding of its role in key physiological processes such as metabolism, immune function, and inflammatory regulation [9]. Beyond maintaining health, the gut microbiota is now recognized as a critical factor in disease progression—including cancer—and in modulating the therapeutic efficacy of pharmacological agents [10,11,12,13].

In healthy adults, the composition of the gut microbiota is relatively stable over time, maintained through complex feedback mechanisms that contribute to intestinal barrier homeostasis. Immune surveillance of microbial communities is continuous and essential for preserving the equilibrium between beneficial and pathogenic species. Disturbances in this equilibrium, commonly referred to as dysbiosis, have been implicated in both gastrointestinal and systemic pathologies [14]. Within this symbiotic environment, the host provides nutrients and habitat, while microbes contribute to host physiology by regulating lipid and glucose metabolism, and supporting immune development through bioactive microbial metabolites [15]. The importance of these interactions is highlighted by studies in germ-free (GF) animals, which demonstrate profound immune and metabolic deficiencies in the absence of a functional microbiota. As our understanding of the gut microbiome deepens, attention has increasingly turned to how pharmaceutical excipients, including CDs, might influence microbial ecology. Emerging research indicates that such interactions may not be benign; rather, they may significantly impact both microbiota composition and host outcomes, warranting careful evaluation in future drug development strategies. This review focuses on the applications and effects of CDs on the gut microbiota.

## 2. Cyclodextrins: General Insights

### 2.1. Structure, Properties, and Chemical Modification

CDs are produced through the enzymatic degradation of starch by cyclodextrin glucosyltransferase. They are a class of cyclic oligosaccharides composed of five or more α-D-glucopyranose units linked by α-(1,4)-glycosidic bonds [16,17]. Among them, β-cyclodextrin (β-CD) is widely used as a pharmaceutical excipient due to its appropriate cavity size, efficient drug-loading capacity, and relatively low cost [18].

Cyclodextrins are physically and chemically stable macromolecules with a truncated cone or toroidal structure, characterized by a hydrophilic exterior and a hydrophobic inner cavity [19,20]. This unique structural feature allows them to interact with both lipophilic and hydrophilic substances, imparting a dual affinity that underpins their pharmaceutical versatility [21]. The hydrophobic cavity enables the encapsulation of drug molecules (guests), forming non-covalent host–guest inclusion complexes that enhance the physical, chemical, and biological properties of the encapsulated agents [20,22,23].

To broaden the applicability of CDs in drug delivery systems, numerous CD derivatives have been developed in parallel with advances in chemical modification techniques. Specific functional groups can be introduced with precision at designated positions, thereby expanding their physicochemical and pharmacological utility. For instance, modified β-CD exhibits greater solubility compared to its unmodified form [24].

Several multifunctional derivatives, such as hydroxypropyl-, sulfobutylether-, and carboxymethyl-β-CDs, have been employed to enhance solubility, stability, and other critical attributes of CD–drug inclusion complexes [25,26]. Among these, hydroxypropyl-β-cyclodextrin (HP-β-CD) and sulfobutylether-β-cyclodextrin (SBE-β-CD) are the most extensively studied and are already registered for use [24]. Additional derivatives, including methylated forms and maltosyl-β-CD (Ma-β-CD), have also found applications in pharmaceutical manufacturing. Hydroxypropyl-γ-CD, in particular, exhibits reduced aggregation compared to unmodified γ-CD. Moreover, certain amphiphilic CD derivatives have been investigated for their enhanced interaction with hydrophobic drugs [27]. Owing to their structural versatility, CDs can also serve as scaffolds for synthesizing polymers with diverse architectures and functionalities, such as CD-centered star polymers, CD-capped polymers, and CD-pendant polymers [28].

### 2.2. Pharmaceutical Uses of CDs

The applications and functions of CDs and their derivatives in various drug delivery systems, including nanospheres, nanosponges, and cyclodextrin based metal organic frameworks, are summarized in Table 1.

## 3. Gut Microbiota

### 3.1. Gut Microbiota: General Insights

The human gut microbiota comprises a consortium of microbes with diverse functions residing in the gastrointestinal tract. This microbial community includes bacteria, archaea, fungi, and viruses, forming a symbiotic relationship with the host [44]. Due to its essential role in immune and physiological regulation, the gut microbiota is often referred to as an “ignored organ”. Bacteria represent the largest component of this community; however, nearly 80% of human-associated bacteria cannot be cultured, and the functions of most remain unknown [45].

Different sections of the gastrointestinal tract host distinct microbial compositions. The colon harbors the highest microbial population, while only a few bacterial species are typically found in the stomach and small intestine. In total, approximately 99% of gut bacteria are anaerobic [46]. Human microbiome projects have identified 2172 microbial species isolated from humans, classified into 12 distinct phyla [46]. At the phylum level, more than 90% of the gut microbiome consists of Firmicutes, Bacteroidetes, and Proteobacteria [47].

The composition of the gut microbiota is dynamic and influenced by multiple environmental factors, including nutrient availability, oxygen concentration or redox state, pH, bile acid concentration, the presence of beneficial or harmful compounds, pressure, and temperature. These variables support the growth of specific microbial populations and determine their activity within the human host [45].

The gut microbiota plays key roles in carbohydrate and protein metabolism, energy production, and the synthesis of cellular components, making it essential to fundamental biological processes. Additionally, it acts as a defensive barrier against environmental threats and constitutes a major component of the gut barrier system [48]. It is also critically involved in immune modulation [49], drug metabolism [50], and the transformation of xenobiotic compounds [51].

The Human Microbiome Project undertook a comprehensive study involving healthy adults from diverse ethnic and gender backgrounds and sampled a range of body sites. The results revealed considerable diversity in both the structure and function of the human microbiome [16]. Despite this interindividual variability, healthy individuals were found to share consistent core metabolic functions within their microbiota [52].

A subset of this research identified three primary gut enterotypes—*Bacteroides*, *Prevotella*, and *Ruminococcus*—each characterized by distinct microbial species and functional capacities. This classification emerged from the analyses of small cohorts across different geographic regions [53]. In summary, the hallmarks of a healthy gut microbiota include high taxonomic diversity, richness in microbial genes, and stable functional output [52].

### 3.2. Gut Microbiota: Influencing Factors

Multiple host-related and environmental factors influence the composition, diversity, and functional dynamics of the gut microbiota, shaping individual microbial signatures and potentially predisposing it to or protecting it from disease.

Ethnicity has been shown to correlate with the presence of specific microbial taxa, suggesting that certain microbial members may mediate health outcomes in a population-specific manner [54]. Meanwhile, the host genotype, including variations related to sex and age, also exerts an independent effect on gut microbial profiles, regardless of ethnic background [55].

Gender-specific differences have been reported as well, with premenopausal women exhibiting a greater microbial diversity and an increased abundance of species associated with improved metabolic health compared to men [56]. Similarly, age has been associated with distinct microbial signatures, even extending to the oral microbiota, indicating that microbial composition evolves throughout the human lifespan [56].

Pregnancy introduces additional variables, as shifts in the vaginal microbiome prior to conception can affect fertility. Moreover, maternal gut microbiota during gestation has been implicated in obstetric outcomes and can exert long-term health effects on both the mother and child [57].

The mode of delivery has a marked influence on neonatal microbial colonization. Vaginally delivered infants are typically colonized by beneficial genera such as *Bacteroides*, *Prevotella*, and *Lactobacillus*, while cesarean-born infants may lack exposure to the maternal vaginal microbiota, instead acquiring *Bacteroides* strains from other sources, such as the maternal gut [58].

Infant feeding practices also shape early microbial establishment. Colonization may begin even before birth, potentially influenced by microbes present in the placenta or amniotic fluid. Breastfeeding promotes the growth of *Lactobacillus* and *Bifidobacterium*, supported by the presence of oligosaccharides in human milk. In contrast, formula-fed infants tend to develop distinct microbial profiles lacking these beneficial taxa [59].

Dietary patterns are among the most powerful modulators of gut microbiota throughout life. While short-term dietary shifts can induce transient microbial changes, the extent to which long-term dietary habits reshape the microbiota in a sustained manner remains an area of active investigation [60].

Additionally, pharmaceutical agents, particularly antibiotics, but also commonly used drugs such as proton-pump inhibitors, metformin, and laxatives, can significantly disrupt microbial homeostasis. These agents may reduce species richness, alter metabolic pathways, and foster the proliferation of antibiotic-resistant strains, thereby increasing the risk of complications such as antibiotic-associated diarrhea or *Clostridioides difficile* infection [61].

Lastly, environmental and lifestyle factors, including exposure to pollutants, socioeconomic conditions, smoking, stress, and diet, have been associated with shifts in microbial composition that may promote chronic inflammation and increase susceptibility to non-communicable diseases [62].

Humans maintain a mutualistic and symbiotic relationship with the trillions of microorganisms that inhabit the gastrointestinal tract. While the host provides a stable environment and a continuous supply of nutrients, the microbiota contributes to numerous physiological functions essential for health. Notably, the dietary choices we make directly shape the composition and functional diversity of these microbial communities [54]. The nutrients we ingest not only nourish the host but also serve as substrates for microbial metabolism, influencing microbial proliferation and activity.

**Figure 2:**
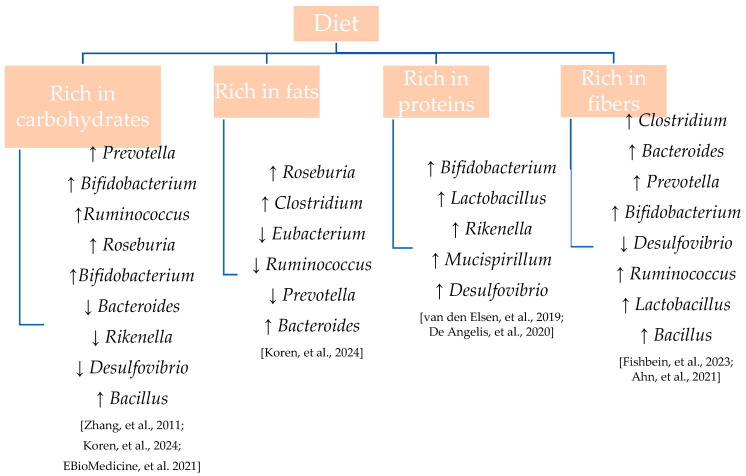
Modulation of gut microbiota genera by different macronutrient profiles.

Evidence suggests that diets rich in saturated fats and animal proteins can disrupt the delicate balance of the gut ecosystem—a condition referred to as dysbiosis. This imbalance has been associated with an increased risk of developing a range of pathologies, including autoimmune diseases, infections, and disorders involving the central nervous system [55]. Conversely, diets high in fiber, polyphenols, and complex carbohydrates are known to promote microbial diversity and enrich beneficial bacterial populations. The impact of various dietary patterns on gut microbiota composition is illustrated in Figure 2 [56,57,58,59,60,61,62].

#### Impact of Prebiotics, Probiotics, Postbiotics, and Synbiotics

Prebiotics and probiotics are increasingly recognized for their central roles in modulating gut microbiota composition and functionality, with significant downstream effects on host physiology and overall health [63]. Probiotics are defined as live microorganisms which, when administered in sufficient quantities, confer health benefits to the host. Among the most studied strains are *Escherichia coli* Nissle 1917, species of the genus Lactobacillus, and *Bifidobacterium*, all of which exert beneficial effects primarily through the production of short-chain fatty acids (SCFAs), including acetate, propionate, and butyrate. These SCFAs are generated by specific bacterial genera such as *Bacteroides* (acetate, propionate), *Faecalibacterium* and *Roseburia* (butyrate), and *Akkermansia* (acetate), and contribute to gut barrier integrity, immune modulation, and pathogen inhibition via competition for nutrients or binding sites along the intestinal epithelium [64]. *Bifidobacterium* not only contributes to gut health and immune regulation but also exhibits antimicrobial activity by inhibiting the colonization of pathogenic bacteria such as *Escherichia coli* and *Salmonella* spp., through mechanisms including competitive exclusion, acidification, and bacteriocin production [65].

Certain commensal bacteria, such as *Bifidobacterium* and *Lactobacilli*, lack lipopolysaccharide structures in their outer membranes, which reduces their pro-inflammatory potential and enhances their safety profile. Moreover, recent findings have identified additional species, including *Roseburia* and *Akkermansia muciniphila*, as promising candidates for next-generation probiotics due to their immunomodulatory and metabolic benefits [66].

In contrast to probiotics, prebiotics are substrates selectively utilized by beneficial host microorganisms that in turn elicit positive effects on health. By supporting the proliferation or metabolic activity of microbial genera such as *Bifidobacterium* and *Lactobacilli*, prebiotics contribute to restoring or maintaining gut microbial equilibrium. Nutritional interventions incorporating prebiotic compounds have demonstrated immunomodulatory activity and the ability to promote intestinal homeostasis [67,68].

More recently, novel prebiotic sources have been explored, particularly those derived from traditional Chinese medicinal fungi. Extracts from *Ganoderma lucidum*, *Hirsutella sinensis*, and *Antrodia cinnamomea* have shown potential not only in promoting beneficial microbial growth, but also in reducing body weight, improving inflammatory status, and mitigating insulin resistance in high-fat diet-induced obesity models [63]. These effects appear to be mediated, at least in part, by microbiota modulation, reinforcing their value as bioactive prebiotic agents for metabolic health enhancement.

Postbiotics, a newer category in microbiome-related therapeutics, are defined as non-viable bacterial components or metabolic byproducts that exert beneficial biological effects on the host [69,70]. Produced during the anaerobic fermentation of substrates such as prebiotics, postbiotics encompass a wide array of functional molecules, including SCFAs, microbial cell wall fragments, extracellular polysaccharides, cell lysates, teichoic acids, vitamins, and other low-molecular-weight metabolites [71].

Unlike live probiotics, postbiotics offer several practical advantages: they are inherently more stable, less prone to contamination, and easier to formulate into various delivery systems. From a functional perspective, postbiotics have demonstrated the ability to enhance gut barrier function, regulate immune responses, and suppress the growth of harmful bacteria [72]. These features position them as promising tools in the management of inflammation-driven and metabolic disorders.

Beyond their physiological functions, postbiotics also have applications in food technology and pharmaceuticals. They are being investigated as natural biopreservatives, components in antimicrobial packaging, and agents for the biodegradation of foodborne contaminants. Pharmacologically, postbiotics exhibit a broad spectrum of bioactivities, including anti-inflammatory, immunomodulatory, antihypertensive, and antioxidant effects.

Synbiotics—combinations of prebiotics and probiotics—are designed to work synergistically to enhance the growth and activity of beneficial microbes while promoting a more resilient and balanced gut ecosystem. These formulations can reinforce host immune responses and inhibit the proliferation of pathogenic organisms [73,74]. Synbiotics have been shown to reduce inflammatory markers, improve insulin sensitivity, and stimulate the release of gut hormones such as glucagon-like peptide-1 (GLP-1), which plays a key role in glucose metabolism [73].

Together, prebiotics, probiotics, postbiotics, and synbiotics—alongside emerging terms such as nutribiotics and pharmabiotics—form a continuum of microbiota-targeted strategies aimed at optimizing host health [75]. By selectively enriching health-promoting microbial populations and suppressing pathogenic species, these compounds hold significant therapeutic potential in immune regulation, metabolic control, and disease prevention. Nonetheless, further mechanistic studies and clinical validation are needed to refine their use and maximize their efficacy in personalized healthcare.

### 3.3. Gut Microbiota: Functions and Impact on Host Health

The gut microbiota plays a pivotal role in human health by regulating a wide spectrum of physiological processes and contributing to the prevention of disease onset. The intricate, bidirectional interactions between the host and intestinal microbial communities have profound implications for systemic health, influencing the development and progression of numerous pathological conditions.

One of the key functions of the gut microbiota lies in its ability to modulate the immune system. Through continuous interaction with mucosal surfaces and systemic immune cells, gut microbes guide the maturation, differentiation, and functional calibration of immune responses [76]. In parallel, they assist in the digestion and absorption of complex dietary components and play a vital role in the endogenous production of micronutrients, including vitamin B12, folic acid, and vitamin K—cofactors essential for metabolic and hematological balance [77].

In addition to their metabolic contributions, commensal microorganisms provide colonization resistance against pathogens by competing for ecological niches, producing antimicrobial peptides, and activating host immune defenses [78]. These protective mechanisms are critical not only for maintaining intestinal integrity but also for influencing metabolic health. Indeed, the gut microbiota modulates host metabolism through its involvement in nutrient assimilation and the regulation of hormones implicated in satiety, energy storage, and adiposity, such as ghrelin, leptin, and peptide YY [79]. Microbial fermentation products, particularly SCFAs and biotransformed polyphenols, exert notable anti-inflammatory and antioxidant effects. These metabolites act as signaling molecules that support intestinal barrier integrity, suppress low-grade inflammation, and reduce oxidative stress, collectively offering protection against chronic disease states such as inflammatory bowel disease, metabolic syndrome, and type 2 diabetes [80].

**Figure 3:**
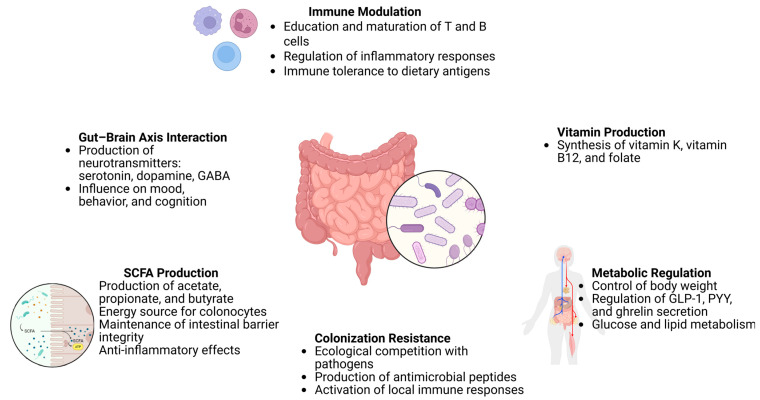
Functions of the gut microbiota and its impact on host health, https://app.biorender.com/illustrations/680cb8a2850813bda4eff5fe (accessed on 14 May 2025).

Overall, the crosstalk between the gut microbiota and key physiological systems, including the immune, nervous, and endocrine networks, is fundamental to the maintenance of homeostasis and the promotion of long-term health (Table 2). Ongoing research continues to unravel the complexity of these interconnections, underscoring the therapeutic potential of microbiota-targeted strategies in precision medicine (Figure 3).

### 3.4. Gut Dysbiosis

Gut dysbiosis, resulting from an imbalance in the gut microbiome, can lead to the excessive production of reactive oxygen species (ROS), promoting inflammation through disruption of gut barrier integrity, activation of the immune system, and alterations in metabolic pathways. As gut dysbiosis alters microbial metabolites, some of which may induce inflammation, ROS production, and epigenetic modifications [80], inflammation-induced ROS can, in turn, exacerbate gut dysbiosis [85,86].

Experimental studies in mice have demonstrated that host-derived ROS significantly alter microbial species diversity and the overall composition of the gut microbiota [86]. In contrast, probiotics that alleviate dysbiosis have been shown to reduce ROS production in the gut, thereby mitigating ROS-induced inflammation [87,88].

Similarly, dietary polyphenols can suppress dysbiosis by scavenging ROS and increasing the abundance of beneficial bacteria such as *Akkermansia muciniphila*, a species typically diminished in obese mice. Higher levels of this bacterium are associated with reduced extracellular ROS concentrations in the gut. Among various antioxidants, including vitamin C, β-carotene, and grape polyphenols, the latter has demonstrated the greatest efficacy in reducing gut ROS and promoting *Akkermansia muciniphila* growth, likely due to its lower systemic bioavailability [89].

It is also important to recognize that, despite their harmful effects under pathological conditions, ROS play a physiological role in stem cell proliferation within colonic epithelial tissue. In this potentially evolutionarily adaptive mechanism, the gut microbiota activates toll-like receptors, which induce NOX1 expression and ROS production, subsequently activating epidermal growth factor receptor signaling and promoting epithelial cell proliferation to maintain colonic homeostasis [90].

## 4. Methods

This review was conducted as a narrative literature review with elements of a systematic search, based on selected aspects of the PRISMA (Preferred Reporting Items for Systematic Reviews and Meta-Analyses) guidelines. We performed searches across Google Scholar, PubMed, and Science Direct using combinations of the following keywords: “cyclodextrin”, “gut”, “gut microbiota”, “prebiotic”, and “probiotic”. Priority was given to studies published in the last five years, particularly in the past year; however, older studies were also considered when recent evidence was lacking. Eligible articles were required to address at least one of the following: (i) structure, properties, chemical modifications, or pharmaceutical applications of cyclodextrins; (ii) gut microbiota composition, functions, influencing factors, or dysbiosis; or (iii) the modulatory effects of cyclodextrins on gut microbial communities. We included full-text articles available in English and excluded abstracts, commentaries, and studies not directly relevant to the research questions. The goal of this narrative synthesis was to map the current landscape and highlight relevant experimental findings rather than perform a quantitative meta-analysis or formal bias assessment.

## 5. Effects of CDs on Gut Microbiota

### 5.1. Effects of CDs on Digestion

Different studies have demonstrated that α-cyclodextrins, when used in drug delivery systems, can also influence digestive processes. Lee et al. found that administration of 1 g/kg α-cyclodextrin exhibited a notable antihyperglycemic effect without stimulating glucagon-like peptide 1 (GLP-1) secretion or delaying intestinal transit. These findings suggest that 1 g/kg α-cyclodextrin may improve hyperglycemia through a GLP-1-independent mechanism. Interestingly, this dose did not enhance insulin secretion, indicating that the glucose-lowering effect is mediated through an insulin-independent pathway.

A higher dose, 2 g/kg α-cyclodextrin, also suppressed hyperglycemia but was associated with increased GLP-1 secretion, unlike the lower dose [91]. Similar outcomes were reported by Wittowski [92], whose meta-analysis showed that α-cyclodextrin administration reduces postprandial glycemic responses. However, the absence of a corresponding increase in insulin levels supports the notion that α-cyclodextrin exerts its beneficial effects independently of insulin production, and thus, its efficacy is not impaired by insulin resistance.

Cyclodextrins have also been shown to bind dietary fats, potentially contributing to reduced cholesterol levels. In an in vivo study involving high-fat diet (HFD) mice, treatment with β-cyclodextrins significantly decreased serum total cholesterol (TC), triglycerides (TG), and low-density lipoprotein cholesterol (LDL-C) levels [93]. Moreover, administration of (-)-epigallocatechin-3-gallate (EGCG) loaded into β-cyclodextrin nanoparticles (EGCG-β-CD NPs) led to greater reductions in serum TC, LDL-C, and phospholipids (PL) compared to EGCG alone. Notably, EGCG-β-CD NPs also markedly attenuated the HFD-induced increase in hepatic TC levels [94].

Another study observed that epididymal adipose tissue weight in the HFD plus α-cyclodextrin group was significantly lower than in the untreated HFD group. Histological analysis further revealed that adipocyte size, which was enlarged in HFD-fed mice, was normalized by α-cyclodextrin supplementation. Additionally, mice in the α-cyclodextrin group exhibited significantly reduced fecal TG levels compared to the HFD group [95].

Furthermore, α-cyclodextrin was shown to promote cholesterol excretion by binding to bile acids, which play a central role in cholesterol metabolism, thereby contributing to decreased circulating cholesterol levels [96]. Beyond cholesterol metabolism, bile acids also function as signaling molecules that regulate lipid and glucose homeostasis through receptors such as FXR (farnesoid X receptor) and TGR5 (G-protein-coupled bile acid receptor 1). By binding bile acids and reducing their reabsorption, α- and β-cyclodextrins may indirectly influence these signaling pathways. FXR activation has been associated with improved insulin sensitivity and reduced hepatic lipogenesis, while TGR5 activation promotes energy expenditure and GLP-1 secretion. These mechanisms support the hypothesis that CDs could contribute to anti-obesity effects not only via microbiota modulation but also through bile acid-mediated metabolic control [97,98,99].

### 5.2. Interaction of CDs with Intestinal Flora

Native cyclodextrins, due to their resistance to digestive enzymes, are only minimally absorbed in the small intestine and pass largely unchanged into the colon, where they become substrates for microbial fermentation. Approximately 60–70% of ingested β-cyclodextrin may reach the large intestine intact. Once there, specific microbial taxa, including *Bacteroides* and *Bifidobacterium* spp., are capable of enzymatically degrading CDs, liberating fermentable oligosaccharides and generating short-chain fatty acids (SCFAs). Chemical modifications can influence this process: for instance, hydroxypropylated and methylated derivatives are more water-soluble and may exhibit altered transit profiles, but often retain resistance to small intestinal digestion. The resulting variability in colonic availability is a critical determinant of their prebiotic potential and microbiota-modulating effects [7,100,101,102]

A cross-sectional study of Japanese long-distance runners and age- and sex-matched nonathletes found that supplementation with (α-CD) in human males increased the abundance of *Bacteroides uniformis* in the gut. This study showed that α-CD, a preferred substrate for *B. uniformis*, elevated its abundance and improved both human and mouse endurance performance [103].

In another study, Zhu et al. reported that cyclodextrin supplementation, particularly β-CD, significantly increased the relative abundance of *Bacteroidetes* and *Verrucomicrobia*, while decreasing the relative abundance of *Firmicutes*. The Firmicutes to Bacteroidetes (F/B) ratio was markedly higher in the HFD baseline group compared to other groups, but cellulose and cyclodextrin supplementation reduced this ratio to levels comparable to those in the control group. Furthermore, cyclodextrin supplementation significantly increased the relative abundance of *Lactobacillus* and *Akkermansia*, an effect not observed with cellulose. Cyclodextrins also downregulated *Allobaculum* and *Ruminococcus* compared to cellulose. The impact of CD supplementation varied depending on the type of cyclodextrin: α-CD reduced the abundance of *Bifidobacterium*, whereas diets containing β-CD and γ-CD had the opposite effect. HFD supplemented with α-CD increased *Lactobacillus* abundance, while β-CD decreased the abundance of *Allobaculum* [93].

Chen et al. found that, at the phylum level, the most abundant bacterial groups included *Firmicutes*, *Bacteroidetes*, *Verrucomicrobia*, and *Proteobacteria*. After 8 weeks of intervention with EGCG–β-CD nanoparticles (EGCG–β-CD NPs), the relative abundance of *Verrucomicrobia* and *Tenericutes* significantly increased, whereas *Bacteroidetes* showed a declining trend. EGCG–β-CD NPs were found to actively modulate *Clostridiales* and *Bacteroidales*. After 8 weeks, the relative abundance of *Verrucomicrobia*, *Lactobacillales*, and *Eggerthellales* increased, while that of *Bacteroidales* and *Enterobacterales* decreased. Moreover, *Caudovirales* and *Mycoplasmatales* were also affected, consistent with the broader shifts in intestinal microbial abundance.

At the family level, the dominant bacterial taxa were *Lachnospiraceae*, *Ruminococcaceae*, *Bacteroidaceae*, and *Akkermansiaceae*, with *Lachnospiraceae* showing the highest relative abundance (17.21%). Other groups such as *Ruminococcaceae* and *Akkermansiaceae* were also prevalent, while *Bacteroidaceae*, *Tannerellaceae*, *Eubacteriaceae*, and *Prevotellaceae* experienced sharp declines. There were minimal differences between groups in the abundance of *Lachnospiraceae*, *Clostridiaceae*, *Oscillospiraceae*, and *Desulfovibrionaceae*. The EGCG–β-CD NPs group was characterized by increased levels of *Akkermansia muciniphila* and decreased levels of *Lachnospiraceae bacterium 28-4*, *Bacteroides sartorii*, and *Oscillibacter* species [93].

**Figure 4:**
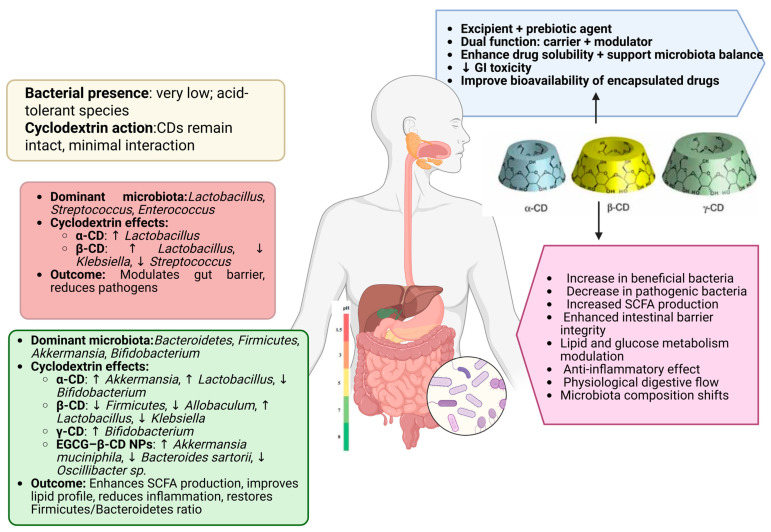
Site-specific gut microbiota composition and CD action, https://app.biorender.com/illustrations/68107683d220218d486a089d (accessed on 17 May 2025).

Wu et al. [104] reported that, at the phylum level, the gut microbial composition across all experimental groups was dominated by four major phyla, *Proteobacteria*, *Firmicutes*, *Actinobacteria*, and *Bacteroidetes*, which together accounted for over 90% of the total microbiota. Notably, the group treated with PTS/β-cyclodextrin (PTS/β-CD) exhibited a higher relative abundance of Firmicutes and Actinobacteria and a marked reduction in Proteobacteria compared to the group treated with β-CD alone. Within these phyla, *Bifidobacterium* was the predominant genus among Actinobacteria, while *Lactobacillus*, a key probiotic taxon, was prominent within Firmicutes. In contrast, many species within the Proteobacteria phylum, such as *Escherichia coli* and *Klebsiella*, are well-known human pathogens (Figure 4).

After 24 h of in vitro fermentation, both the β-CD and PTS/β-CD groups demonstrated a decrease in Bacteroides abundance compared to the untreated (blank) group. This shift may be attributable to pH changes induced during fermentation, which can alter microbial viability and competitiveness. At the genus level, significant increases in beneficial genera such as *Bifidobacterium* and *Lactobacillus* were observed in the PTS/β-CD group, accompanied by a notable suppression of opportunistic bacteria, including *Lactococcus*, *Streptococcus*, and *Klebsiella*. Similarly, in the β-CD group, an increase in *Lactobacillus* and a decrease in *Klebsiella* were reported compared to the blank control.

Collectively, these findings suggest that β-CD exerts dual functionality when used as a carrier for bioactive compounds like PTS: it not only enhances delivery through encapsulation but also acts as a prebiotic, favorably modulating gut microbiota composition and supporting gut barrier integrity (Table 3) [104].

### 5.3. CDs and SCFAs

SCFAs are the main metabolites produced by the intestinal microbial community. They not only serve as an important energy source for intestinal epithelial cells but also play a key role in regulating the intestinal microenvironment, suppressing inflammatory responses, and maintaining the integrity of the gut barrier [105].

Zhu et al. reported that supplementation with α-CD and γ-CD significantly increased total SCFA concentrations in the cecum of mice fed a high-fat diet. The addition of 6% γ-CD raised SCFA levels by approximately 1.44-fold, showing greater efficacy than cellulose. This effect was largely attributed to elevated levels of acetate and propionate. While α-CD supplementation significantly reduced isobutyrate concentrations, it had no notable effect on butyrate levels. β-CD supplementation had a minimal impact on SCFA concentrations, with the exception of an increase in isovalerate levels [93].

Another study involving in vitro probiotic experiments showed that, compared to the β-CD and fructooligosaccharide (FOS) groups, the PTS/β-CD complex significantly enhanced the production of acetic acid, butyric acid, and lactic acid within 24 h. Additionally, relative to the blank group, the β-CD group demonstrated significantly higher levels of propionic and butyric acids [104].

Regarding α-CD, a separate study found that mice in the HFD plus α-CD group exhibited a significant increase in total SCFA content in the cecum compared to the HFD group alone. When combined with anthocyanins, acetic acid was the predominant SCFA, followed by propionic acid. No significant differences were observed across time points, and only minor differences emerged when comparing treatment groups. SCFA, lactic acid, and formic acid concentrations remained consistent between treatments from baseline to 8 h, with notable changes only observed after 24 h of incubation [106].

### 5.4. Toxicological Implications of CDs

While cyclodextrins are generally recognized as safe, particularly for oral administration, their safety profile is highly dependent on the specific type of cyclodextrin, route of administration, dosage, and the guest molecules with which they form inclusion complexes. Naturally occurring forms, such as α-, β-, and γ-cyclodextrin, exhibit favorable safety characteristics, especially when administered orally. For oral use, native cyclodextrins tend to have low toxicity, as they are poorly absorbed in the gastrointestinal tract and are primarily excreted unchanged in the feces. For example, γ-cyclodextrin has been granted generally recognized as safe (GRAS) status by regulatory authorities for use in both food products and pharmaceuticals [107].

However, safety concerns may arise when cyclodextrins are administered through non-oral routes, such as parenterally—via intravenous or intramuscular injection. β-cyclodextrin, in particular, has been associated with nephrotoxicity following parenteral administration, especially at high doses [108]. This toxicity is largely attributed to its accumulation in renal tissue, where it may damage kidney cells. Furthermore, β-cyclodextrin is known to induce hemolysis, particularly when administered intravenously or at high concentrations. This effect is due to its capacity to extract cholesterol and phospholipids from cellular membranes, resulting in membrane destabilization and cell lysis [109].

Such limitations have led to the development of safer derivatives, such as hydroxypropyl-β-cyclodextrin HP-β-CD, which demonstrates reduced toxicity and improved renal tolerance [110]. HP-β-CD also exhibits high osmotic activity and undergoes partial degradation by gut microbiota. Nevertheless, in drug delivery systems, variability in the release kinetics of inclusion complexes can pose challenges for dosing precision and therapeutic predictability. In acidic environments such as the stomach, these complexes may dissociate more rapidly, leading to premature drug release and potential local irritation or toxicity. Conversely, in neutral or basic environments, drug release may be delayed, potentially reducing therapeutic efficacy or resulting in uneven drug distribution [111].

The type of cyclodextrin used, its degree of substitution, and the physicochemical nature of the inclusion complex all play critical roles in determining the safety and efficacy of cyclodextrin-based formulations. Through continued research and rigorous evaluation, the safe and effective use of cyclodextrins across various applications can be optimized, ensuring that their therapeutic benefits are maximized while minimizing potential risks.

## 6. Future Perspectives

Looking forward, it will be essential to clarify the mechanisms through which different types of CDs and their derivatives interact with specific microbial enzymes, receptors, and transport systems. Future research should explore how inclusion complexes release their active compounds in the gut environment and how CDs themselves are metabolized by gut microbes. Multi-omics strategies, integrating metagenomics, metabolomics, and transcriptomics, hold great promise for elucidating the dynamic interplay between CDs, microbiota, and host physiology. These approaches could reveal how targeted CD formulations can be tailored to deliver not only pharmacological benefits but also prebiotic effects that support gastrointestinal health.

Next-generation CD-based systems may purposefully leverage these microbiome-mediated interactions to enhance therapeutic efficacy and reduce toxicity. Incorporating microbiome-aware evaluations into CD development pipelines will be crucial for optimizing drug delivery platforms and unlocking new applications in nutraceuticals and therapeutic agents that actively contribute to maintaining or restoring gut homeostasis.

Furthermore, future investigations should explore the role of CDs in modulating microbiota–host interactions under environmental and epigenetic pressures, including those induced by climate change. The application of high-throughput sequencing platforms, such as NGS and WGS, will be instrumental in identifying previously uncultivable bacteria and fungi. In addition, the emerging concept of the gut–brain axis, particularly the potential of bacterial metabolites to cross the blood–brain barrier (BBB), represents a fertile area for CD-based interventions aimed at neuroinflammation and neurodegenerative disorders.

## 7. Limitations

Although the review incorporated a structured search strategy across multiple databases and followed selected principles of PRISMA guidelines, it was ultimately conducted as a narrative literature review rather than a full systematic review.

As such, the number of records screened, full texts assessed, and reasons for exclusion were not formally recorded.

Potential biases in article selection may exist due to subjective judgment during eligibility assessment. Additionally, the lack of quantitative synthesis and meta-analysis limits the ability to draw definitive comparative conclusions between different cyclodextrins and their modulatory effects on the gut microbiota.

Future systematic reviews and meta-analyses with rigorous study selection processes are warranted to further validate and quantify these findings.

## 8. Conclusions

CDs represent a unique class of molecules with multifaceted applications, extending beyond pharmaceutical excipients to modulators of the gut microbiota. Their influence on the abundance of beneficial microbial genera, SCFA production, and restoration of intestinal eubiosis highlights their emerging prebiotic-like roles. By selectively enriching *Bifidobacterium*, *Lactobacillus*, and *Akkermansia*, CDs may contribute positively to metabolic and immune-related health outcomes.

While current evidence supports their promising effects, the majority of findings stem from preclinical models. Further research, especially well-designed human clinical trials, is essential to clarify the safety, efficacy, and mechanistic underpinnings of CDs in gut microbiota modulation. Additionally, exploring their synergy with dietary patterns and probiotic strategies could unlock new avenues for therapeutic interventions.

## Figures and Tables

**Table 1:** Pharmaceutical uses of CDs.

Drug-Delivery System.	CD Type	Active Substance	Effect	Ref.
Emulsion	γ-CD/sodium caseinate/alginate (Alg)	Curcumin	Demonstrated excellent stability in highly acidic (pH 3.0), alkaline (pH 11.0), and thermal (90 °C) conditions.	[29]
	α-CDs with octenylsuccinic anhydride (OSA)	Curcumin	Maintained stability over 30 days. Smaller particles released more fatty acids. Bioavailability rose by 10.3%.	[30]
Liposome	E-βCD/D-βCD/βCD	Curcumin	Showed over fivefold higher encapsulation efficiency than conventional liposomes.	[31]
	HP-β-CD	Brinzolamide (BRZ)	Achieved entrapment efficiency of 92.50 ± 2.10%.	[32]
MOFs	γ-CD	Paeonol (PAE)	CD-based MOFs increased permeability by five times versus free PAE.	[33]
	γ-CD	Honokiol (HNK)	Enhanced both solubility and dissolution rate of the drug.	[34]
Nanosponge	β-CD-CMC-g poly	Docetaxel	Improved aqueous solubility up to 14-fold.	[35]
	β-CD	Tapentadol	Released 51.62–82.34% of the drug over 6 h, with enhanced control.	[36]
Nanospheres	HP-β-CD	Idebenone (IDE)	Facilitated stronger permeation and interaction than unencapsulated IDE.	[37]
	α- and β-CD	Erlotinib (ERL)	Boosted anticancer potential in standard and 3D models of lung and liver tumors.	[30]
Nanoparticles	HP-CD	Meropenem	Notably increased drug solubility in water-based media.	[38]
	Mannose-modified γ-CD	Regorafenib (RG)	Advanced both pharmacokinetic profile and drug performance.	[39]
	6-O-capro-β-CD and PC β-CDC	Paclitaxel	Strengthened antitumor activity.	[40]
Nanogel	HP-β-CD	Dexibuprofen	Porous, amorphous design allowed for significantly improved release and good compatibility.	[41]
	β-CD conjugated hyaluronic acid (HA-β-CD)	Small molecules and proteins	Designed as a robust and adaptable delivery platform.	[42]
	β-CD	Methotrexate (MTX) and doxorubicin (DOX)	Exhibited dual responsiveness to pH and temperature and showed photoluminescence.	[43]

**Table 2:** Aiding microbes in the secretion of gut hormones, NK-not known.

Aiding Microbes	Gut Hormones	Secretory Cells	Ref.
*Bifidobacterium*, *Lactobacillus*, *Akkermansia muciniphila*	Glucagon-like peptide 1	Colonic L-cells	[81]
*Bifidobacterium*, *Lactobacillus*, *Akkermansia muciniphila*, *Escherichia*, *Enterococcus*, *Trichuris*	Peptide YY	Colonic L-cells	[81]
*Prevotella*, *Lactobacillus*	DPP 4	Enterocytes, epithelial cells, immune cells	[82]
NK	Ghrelin	Cardiomyocytes	[83]
NK	Oxytomodulin	Pancreatic cells	[83]
NK	Neurotensin	Gastrointestinal endocrine N-cells	[83]
NK	Motilin	Endocrine M-cells	[83]
*Escherichia*, *Bacillus*, *Saccharomyces*	Dopamine or noradrenaline	Nerve cells	[84]
NK	Acetylcholine	Nerve cells	[84]
*Lactobacillus*, *Bifidobacterium*	GABA	B-cells	[84]
*Lactobacillus*, *Clostridium*	Indole	NK	[84]
*Acetatifactor*, *Bacteroides*	Bile acids	Hepatocytes	[84]
*Clostridium* spp., *Escherichia*, *Enterococcus*, *Faecalibacterium*, *Candida*, *Streptococcus*	Serotonin	Enterochromaffin cells	[84]

**Table 3:** Effects of cyclodextrins and CD-based formulations on gut microbiota composition at the taxon and phylum level.

Study (CD Type)	Taxon	Change	Study (CD Type)	Phylum	Change
Zhu et al. (all CDs)	*Lactobacillus*	↑	Zhu et al. (α-CD)	*Bacteroidetes*	↑
	*Akkermansia*	↑		*Verrucomicrobia*	↑
	*Allobaculum*	↓		*Firmicutes*	↓
	*Ruminococcus*	↓		F/B ratio	↓ (toward control)
Zhu et al. (α-CD)	*Lactobacillus*	↑		*Bifidobacterium*	—
	*Bifidobacterium*	↓	Zhu et al. (β-CD)	*Bacteroidetes*	—
Zhu et al. (β-CD)	*Allobaculum*	↓		*Verrucomicrobia*	—
Zhu et al. (γ-CD)	*Bifidobacterium*	↑		*Firmicutes*	↓ (toward control)
Chen et al. (EGCG-β-CD NPs)	*Lactobacillales* (order)	↑		F/B ratio	↓
	*Eggerthellales* (order)	↑	Zhu et al. (γ-CD)	*Bifidobacterium*	↑
	*Bacteroidales* (order)	↓	Chen et al. (EGCG-β-CD NPs)	*Verrucomicrobia*	↑
	*Enterobacterales* (order)	↓		*Tenericutes*	↑
	*Lachnospiraceae bacterium* 28–4 (species)	↓		*Bacteroidetes*	↓
	*Bacteroides sartorii* (species)	↓	Wu et al. (PTS/β-CD)	*Actinobacteria*	↑
	*Oscillibacter* sp. (species)	↓		*Firmicutes*	↑
	*Akkermansia muciniphila* (species)	↑		*Proteobacteria*	↓
Wu et al. (PTS/β-CD)	*Bifidobacterium*	↑	Wu et al. (β-CD)	*Bacteroides*	↓
	*Lactobacillus*	↑			
	*Lactococcus*	↓			
	*Streptococcus*	↓			
	*Klebsiella*	↓			
**Wu et al.** (β-CD)	*Lactobacillus*	↑			
	*Klebsiella*	↓			

## Data Availability

Data are contained within the article.
